# Crosstalk Influence between P38MAPK and Autophagy on Mitochondria-Mediated Apoptosis Induced by Anti-Fas Antibody/Actinomycin D in Human Hepatoma Bel-7402 Cells

**DOI:** 10.3390/molecules22101705

**Published:** 2017-10-17

**Authors:** Yu Wang, Chunhui Xia, Yanxin Lv, Chengjun Li, Qingbu Mei, Hongmei Li, Haijun Wang, Shuang Li

**Affiliations:** 1 Basic Medicine Department, Qiqihar Medical University, Qiqihar 161006, China; wyfr1970@126.com (Y.W.); lv_yanxin@yahoo.com.cn (Y.L.); lichengjun@tom.com (C.L.); mei123456@tom.com (Q.M.); 2 Pharmacy Department, Qiqihar Medical University, Qiqihar 161006, China; lihongmei1969@sina.com (H.L.); wanghaijun0417@yahoo.com.cn (H.W.); lishuang@163.com (S.L.)

**Keywords:** anti-Fas antibody/actinomycin D, P38MAPK, autophagy, mitochondria, apoptosis

## Abstract

Our previous study indicated that anti-Fas antibody/actinomycin D (AF/AD) induced apoptosis of human hepatocellular carcinoma Bel-7402 cells; however, crosstalk influence between P38MAPK and autophagy on mitochondria-mediated apoptosis induced by AF/AD in Bel-7402 cells remains unclear. Therefore, effect of AF/AD on apoptosis, autophagy, phosphorylated-P38MAPK (p-P38MAPK), and membrane potential (ΔΨm) with or without the P38MAPK inhibitor SB203580 or the autophagy inhibitor 3-methyladenine (3-MA) in Bel-7402 cells was investigated in the present study. The results showed that AF/AD resulted in induction of apoptosis concomitant with autophagy, upregulation of p-P38MAPK and autophagy-associated gene proteins (Atg5-Atg12 protein complex, Atg7, Atg10, Beclin-1, LC3 I, and LC3 II), and downregulation of ΔΨm in Bel-7402 cells. In contrast, SB203580 attenuated the effects of AF/AD in Bel-7402 cells. Furthermore, the findings also demonstrated that 3-MA inhibited the impact of AF/AD on autophagy, Atg5-Atg12 protein complex, Atg7, Atg10, Beclin-1, LC3 I, LC3 II, and ΔΨm, and promoted the influence of AF/AD on apoptosis and p-P38MAPK in Bel-7402 cells. Taken together, we conclude that crosstalk between P38MAPK and autophagy regulates mitochondria-mediated apoptosis induced by AF/AD in Bel-7402 cells.

## 1. Introduction

Apoptosis (Type I programmed cell death) and autophagy (Type II programmed cell death) are discrete cellular processes that play a crucial role during development and maintenance of tissue homeostasis. Recent studies have suggested that apoptosis and autophagy are two key pathways in treatment process of anticancer drugs [1,2]. The mitochondrion is a membrane-enclosed organelle occurring in eukaryotic cells. Accumulating evidence has demonstrated that mitochondrial membrane permeabilization is a key event in drugs-induced apoptosis and autophagy of cancer cells [3,4,5]. P38 mitogen-activated protein kinase (MAPK), a member of the MAPK family, plays a key regulatory role in cell proliferation, apoptosis, and autophagy. Past reports have shown that P38MAPK is a major determinant of the balance between apoptosis and autophagy triggered by drugs in cancer cells [6,7].

Anti-Fas antibody (AF) is a 40-kDa type II transmembrane protein belonging to the tumor necrosis factor family. When AF binds to a Fas receptor, it can trigger cells apoptosis [8,9,10]. Furthermore, actinomycin D (AD), a chemotherapy drug used in treatment of a variety of cancers, is capable of inducing cells apoptosis through binding to DNA [11,12,13].

Certain studies have revealed that FA/AD can apparently induce apoptosis in some cells [14,15,16]. Our previous research showed that p38MAPK regulated caspase-3 in AF/AD-induced apoptosis of human hepatocellular carcinoma Bel-7402 cells [17]. However, mechanisms of AF/AD-induced apoptosis of Bel-7402 cells have not been fully clarified. The aim of this study was to investigate crosstalk influence between P38MAPK and autophagy on mitochondria-mediated apoptosis induced by AF/AD in Bel-7402 cells. The results indicate that crosstalk between P38MAPK and autophagy might regulate mitochondria-mediated apoptosis induced by AF/AD in Bel-7402 cells.

## 2. Results

### 2.1. AF/AD Induces Apoptosis Concomitant with Autophagy in Bel-7402 Cells

**Figure 1:**
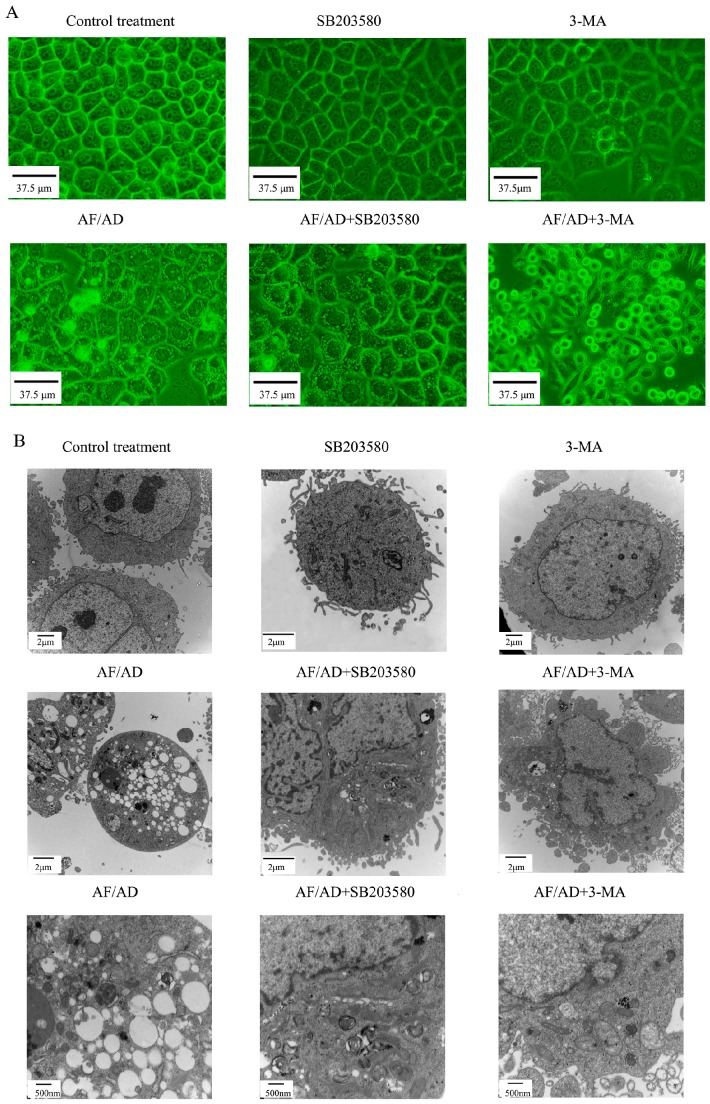
Effect of AF/AD on Bel-7402 cells morphology with or without SB203580 or 3-MA. Bel-7402 cells were pretreated with AF (6 μM)/AD (20 μM) in the absence or presence of SB203580 (10 μM) or 3-MA (5 mM) for 24 h, and then cell morphology was analyzed by inverted microscope (**A**) and transmission electron microscopy (**B**).

**Figure 2:**
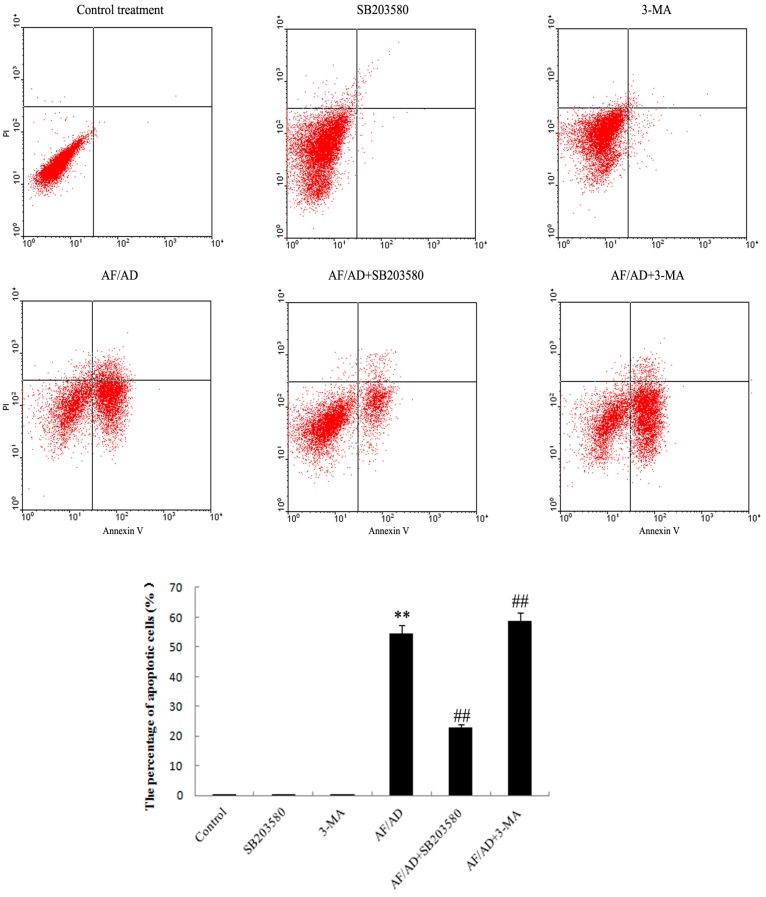
Effect of AF/AD on apoptosis of Bel-7402 cells with or without SB203580 or 3-MA. Bel-7402 cells were pretreated with AF (6 μM)/AD (20 μM) in the absence or presence of SB203580 (10 μM) or 3-MA (5 mM) for 24 h, and then the percentage of apoptotic cells was evaluated by flow cytometry analysis of annexin V-FLUOS/PI double-stained cells. Values presented are representative of three independent experiments (means ± S.D.; ** *p* < 0.01, compared with control treatment; ^##^ *p* < 0.01, compared with AF/AD treatment).

To evaluate whether AF/AD induced apoptosis concomitant with autophagy in Bel-7402 cells, the occurrence of apoptosis and autophagy was detected by inverted microscope and electron microcopy assay. Compared with the control Bel-7402 cells, AF/AD-treated cells simultaneously exhibited apoptotic and autophagic characteristics such as cell shrinkage, nuclear condensation, and cytoplasmic vacuoles (Figure 1A,B). In addition, apoptosis concomitant with autophagy was further quantitated by flow cytometry analysis of annexin V-FLUOS/PI double-stained cells. Compared with the control treatment, AF/AD significantly led to an increase of apoptotic cell percentages (Figure 2).

**Figure 3:**
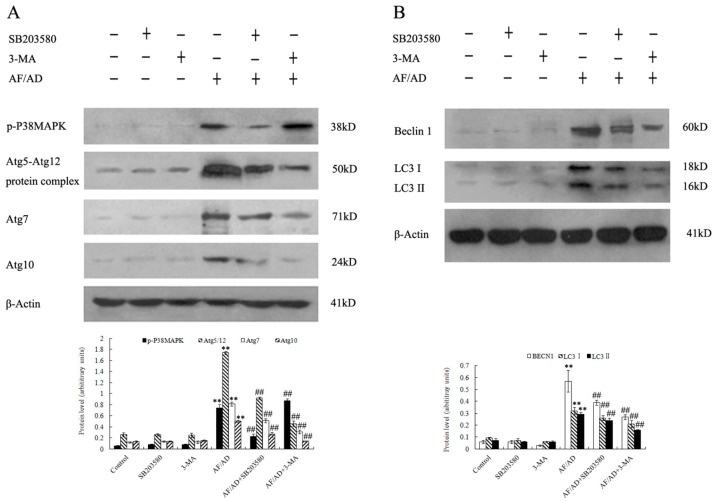
Effect of AF/AD on p-P38MAPK, Atg5-Atg12 protein complex, Atg7, Atg10, Beclin-1, LC3 I, and LC3 II, respectively, with or without SB203580 or 3-MA in Bel-7402 cells analyzed by immunoblot assay. Bel-7402 cells were pretreated with AF (6 μM)/AD (20 μM) in the absence or presence of SB203580 (10 μM) or 3-MA (5 mM) for 24 h. Expression of p-P38MAPK, Atg5-Atg12 protein complex, Atg7, and Atg10 (**A**), and expression of Beclin-1 and LC3 (**B**) were analyzed by immunoblot assay. Values presented are representative of three independent experiments (means ± S.D.; ** *p* < 0.01, compared with control treatment; ^##^ *p* < 0.01, compared with AF/AD treatment).

**Figure 4:**
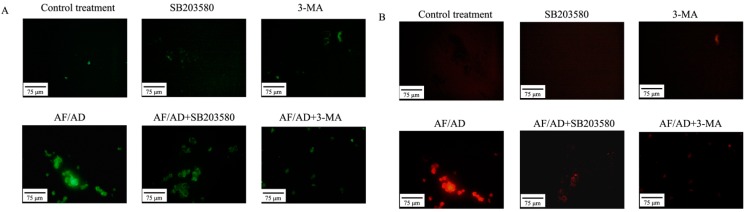
Effect of AF/AD on Beclin-1 and LC3, respectively, with or without SB203580 or 3-MA in Bel-7402 cells analyzed by immunofluorescence assay. Bel-7402 cells were pretreated with AF (6 μM)/AD (20 μM) in the absence or presence of SB203580 (10 μM) or 3-MA (5 mM) for 24 h. Expression of Beclin-1 (**A**) and LC3 (**B**) were analyzed by immunofluorescence assay.

To further ascertain the involvement of autophagic process in AF/AD-induced apoptosis of Bel-7402 cells, the levels of autophagy-associated genes proteins, which are called Atg proteins including Atg5-Atg12 protein complex, Atg7, Atg10, Beclin-1 (Atg-6), and LC3-I/II (Atg-8) was examined by immunoblot and immunofluorescence assay. Compared with the control treatment, AF/AD upregulated expression of Atg5-Atg12 protein complex, Atg7, Atg10, Beclin-1, LC3 I, LC3 II, green Beclin-1 immunofluorescence, and red LC3 immunofluorescence (Figure 3A,B and Figure 4A,B). Furthermore, the autophagy inhibitor 3-methyladenine (3-MA) was applied to block autophagy in Bel-7402 cells. Compared with the AF/AD treatment, 3-MA attenuated the effects of AF/AD on autophagic characteristics, Atg5-Atg12 protein complex, Atg7, Atg10, Beclin-1, LC3 I, and LC3 II (Figure 1A,B, Figure 3A,B and Figure 4A,B).

### 2.2. Autophagy Regulates AF/AD-Induced Apoptosis of Bel-7402 Cells

To determine whether autophagy regulates AF/AD-induced apoptosis of Bel-7402 cells, the effect of 3-MA on apoptosis was tested. Compared with the AF/AD treatment, 3-MA promoted the AF/AD-induced apoptosis of Bel-7402 cells (Figure 1A,B and Figure 2).

### 2.3. P38MAPK Regulates AF/AD-Induced Apoptosis Concomitant with Autophagy in Bel-7402 Cells

To assess whether P38MAPK is involved in AF/AD-induced apoptosis concomitant with autophagy in Bel-7402 cells, the effect of AF/AD on phosphorylated-P38MAPK (p-P38MAPK) with or without the P38MAPK inhibitor SB203580 was investigated by immunoblot assay. Compared with the control treatment, AF/AD activated P38MAPK (Figure 3A); however, compared with the AF/AD treatment, SB203580 reduced the level of p-P38MAPK (Figure 3A). Moreover, SB203580 was used to further firm the regulatory role of P38MAPK during AF/AD-induced apoptosis concomitant with autophagy in Bel-7402 cells. Compared with the AF/AD treatment, SB203580 inhibited the AF/AD-induced apoptosis concomitant with autophagy in Bel-7402 cells (Figure 1A,B and Figure 2).

### 2.4.Crosstalk between P38MAPK and Autophagy Regulates AF/AD-Induced Apoptosis of Bel-7402 Cells

To elucidate whether P38MAPK regulates autophagy, and autophagy in turn regulates P38MAPK, immunoblot and immunofluorescence assay were performed to demonstrate the effect of SB203580 on autophagy, and the effect of 3-MA on P38MAPK during AF/AD-induced apoptosis concomitant with autophagy in Bel-7402 cells. Compared with the AF/AD treatment, 3-MA led to upregulation of p-P38MAPK (Figure 3A), and SB203580 resulted in less morphological characteristics of autophagy (Figure 1A,B), and downregulation of Atg5-Atg12 protein complex, Atg7, Atg10, Beclin-1, LC3 I, LC3 II, green Beclin-1 immunofluorescence, and red LC3 immunofluorescence (Figure 3A,B and Figure 4A,B).

### 2.5. Crosstalk between P38MAPK and Autophagy Regulates Mitochondria in AF/AD-Induced Apoptosis of Bel-7402 Cells

**Figure 5:**
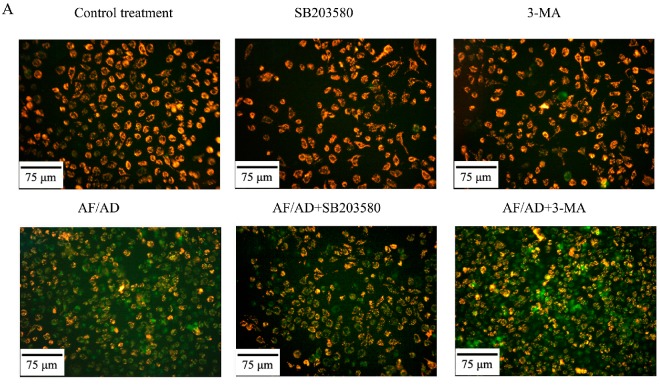

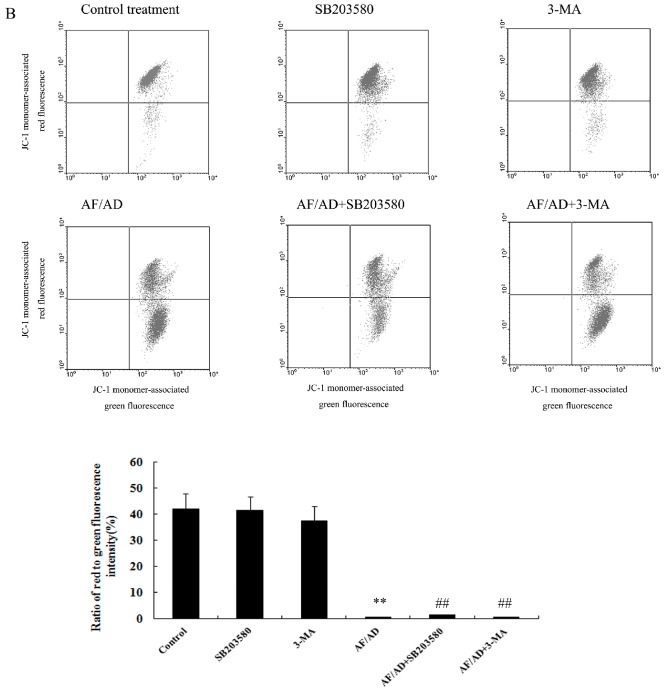
Effect of AF/AD on ΔΨm of Bel-7402 cells with or without SB203580 or 3-MA. Bel-7402 cells were pretreated with AF (6 μM)/AD (20 μM) in the absence or presence of SB203580 (10 μM) or 3-MA (5 mM) for 24 h. Red/green fluorescence intensity was detected by JC-1 assay of fluorescence microscope (**A**), and ratio of red to green fluorescence intensity was analyzed by JC-1 assay of flow cytometry (**B**). Values presented are representative of three independent experiments (means ± S.D.; ** *p* < 0.01, compared with control treatment; ^##^ *p* < 0.01, compared with AF/AD treatment).

To explore the involvement of mitochondria in AF/AD-induced apoptosis and autophagy of Bel-7402 cells, the influence of AF/AD on mitochondrial membrane potential (ΔΨm) was evaluated by JC-I assay of fluorescence microscope and flow cytometry. Compared with the control treatment, AF/AD cause to an increase of apoptotic cells with green fluorescence (Figure 5A), and a decrease in ratio of red/green fluorescence intensity (Figure 5B), indicating that decrease of ΔΨm existed in AF/AD-induced apoptosis and autophagy of Bel-7402 cells. SB203580 and 3-MA were used to further clarify the regulatory effect of P38MAPK and autophagy on ΔΨm in AF/AD-induced apoptosis of Bel-7402 cells. Compared with the AF/AD treatment, SB203580 attenuated the effect of AF/AD on ΔΨm (Figure 5), but 3-MA strengthened the influence of AF/AD on ΔΨm (Figure 5).

## 3. Discussion

Accumulating evidence has suggested that AF/AD induces apoptosis in some cells [14,15,16]. We previously revealed that AF in the presence of AD induced apoptosis of Bel-7402 cells in a dose-dependent pattern [17]. However, whether AF/AD could induce apoptosis concomitant with autophagy in Bel-7402 cells is not ascertained. Therefore, we performed inverted microscope and electron microcopy assay of this, confirming that AF/AD induced apoptosis concomitant with autophagy in Bel-7402 cells.

Autophagic processes start with autophagosome formation which is regulated by Atg proteins. Atg7-mediated formation of two ubiqutin-like Atg protein conjugates, Atg5-Atg12 and LC3-I/II-phosphatidylethanolamine, function at a late step of autophagosome formation [18]. Moreover, Beclin-1and Atg-10 are essential for formation of autophagosomal structures [18]. Our data indicated that AF/AD resulted in upregulation of Atg5-Atg12 protein complex, Atg7, Atg10, Beclin-1, LC3 I, and LC3 II, but that 3-MA attenuated the effects of AF/AD on these autophagy-associated genes proteins in Bel-7402 cells, suggesting that autophagy was involved in AF/AD-induced apoptosis of Bel-7402 cells.

Several reports have revealed that autophagy plays a crucial regulatory role in drug-induced apoptosis [19,20,21]. However, the regulatory effect of autophagy on AF/AD-induced apoptosis of Bel-7402 cells is still not elucidated. Therefore, we studied the influence of 3-MA on AF/AD-induced apoptosis. We found that 3-MA promoted apoptosis, suggesting that autophagy regulated AF/AD-induced apoptosis of Bel-7402 cells.

P38MAPK, which is activated by phosphorylation, has been proven to be a key regulatory protein in induction of apoptosis [17,22,23]. Previous studies have reported that p-P38MAPK is involved in apoptosis concomitant with autophagy in drugs-treated cancer cells [24,25,26,27]. However, the mechanism by which P38MAPK regulates AF/AD-induced apoptosis concomitant with autophagy in Bel-7402 cells is not ascertained. Therefore, we analyzed the effect of AF/AD on p-P38MAPK in combination with SB203580, and the effect of SB203580 on apoptosis concomitant with autophagy in Bel-7402 cells. We observed that AF/AD activated P38MAPK, and that SB203580 reduced the level of activated P38MAPK and protected from AF/AD-induced apoptosis concomitant with autophagy in Bel-7402 cells. These results indicate that activated P38MAPK is essential for AF/AD-induced apoptosis concomitant with autophagy in Bel-7402 cells.

Certain studies have demonstrated that P38MAPK regulates autophagy [20,21,27,28], and autophagy in turn regulates P38MAPK in drugs-induced apoptosis of cancer cells [24,25,26]. However, whether P38MAPK and autophagy regulate each other in AF/AD-induced apoptosis of Bel-7402 cells remains unknown; therefore, the effect of SB203580 on autophagy and the effect of 3-MA on P38MAPK in AF/AD-induced apoptosis of Bel-7402 cells were examined. The results indicated that 3-MA increased the activation of P38MAPK, and that SB203580 caused less morphological characteristics of autophagy, and downregulation of Atg5-Atg12 protein complex, Atg7, Atg10, Beclin-1, LC3 I, and LC3 II. All of the above results indicate that crosstalk between P38MAPK and autophagy regulate AF/AD-induced apoptosis of Bel-7402 cells.

Early reports point out that P38MAPK and autophagy regulate mitochondria in drug-induced apoptosis of cancer cells [22,29,30,31]. However, it is not clear whether P38MAPK and autophagy regulate mitochondria in AF/AD-induced apoptosis of Bel-7402 cells. Therefore, the influence of AF/AD in combination with SB203580 or 3-MA on ΔΨm was investigated. The findings demonstrated that AF/AD resulted in the reduction of ΔΨm, SB203580 attenuated the effect of AF/AD on ΔΨm, and 3-MA strengthened the influence of AF/AD on ΔΨm, suggesting that crosstalk between P38MAPK and autophagy regulated mitochondria in AF/AD-induced apoptosis of Bel-7402 cell.

## 4. Exprimental Section

### 4.1. Materials

Anti-phosphorylated (p)-P38MAPK antibody was purchased from Cell Signaling Technology (Danvers, MA, USA). P38MAPK inhibitor SB 203580 and actinomycin D were purchased from Merck Calbiochem (Darmstadt, Germany). Anti-Beclin-1, anti-LC3, anti-Atg5, anti-Atg7, anti-Atg10, anti-Atg12, agonistic anti-Fas, and anti-β-actin antibodies were purchased from Santa Cruz Biotechnology (Santa Cruz, CA, USA). The Annexin-V-FLUOS Staining kit was purchased from Roche (Basel, Switzerland). The 5,5′,6,6′-tetrachloro-1,1′,3,3′-tetraethylbenzimidazolylcarbocyanine iodide (JC-1) staining kit was purchased from Genmed Scientifics (Wilmington, DE, USA). 3-MA, goat anti-mouse IgG(H+L)-FITC, and rabbit anti-goat IgG(H+L)-TRITC were obtained from Santa Cruz Biotechnology. All other chemicals and reagents were of analytical grade.

### 4.2. Cell Culture and Treatment

Human hepatocellular carcinoma Bel-7402 cells were offered from the Institute of Zoology, Chinese Academy of Science (Beijing, China), and cultured in DMEM medium supplemented containing 10% fetal bovine serum, 100 U/mL penicillin, and 100 μg/mL streptomycin in a humidified incubator with 5.0% CO2 at 37 °C. Cells in logarithmic growth phase were incubated for 24 h at 37 °C, and then treated with AF/AD with or without P38 MAPK inhibitor SB203580 or autophagy inhibitor 3-MA.

### 4.3. Cell Morphology Assay

Cell morphology was assayed using inverted microscope and transmission electron microscope. For inverted microscope analysis, after AF/AD treatment in the absence or presence of SB203580 or 3-MA, cells was observed under a DY5000X inverted microscope (Chongqing Photoelectric Instrument Co. Ltd., Chongqing, China). For transmission electron microscope analysis, after AF/AD treatment in the absence or presence of SB203580 or 3-MA, cells were fixed with 3% glutaraldehyde in 0.1 M sodium cacodylate buffer, transferred to 0.1 M phosphate buffer, and then postfixed with 1% osmium tetroxide in Scollidine. After gradient dehydration in ethanol and acetone, the cells transferred to propylene oxide were embedded in Epon 812. Semi thin sections stained with 1% methylene blue were sectioned into ultrathin slices. Afterward, ultrathin slices were contrasted with uranyl acetate and lead citrate, and detected under a HT7700 transmission electron microscope (Hitachi, Tokyo, Japan).

### 4.4. Annexin V-FLUOS/Propidium Iodide (PI) Double-Staining Analysis of Apoptosis

The Annexin V-FLUOS/PI apoptosis detection kit was used to detect apoptotic cells. Briefly, the harvested cells were resuspended in 100 µL Annexin V binding buffer A. After addition of 2.0 µL Annexin V-FLUOS and 2.0 µL PI, the cell suspension was incubated for 5 min at room temperature in the dark. Afterward, 400 µL binding buffer was added to the cells and 1 × 10^4^ annexin V- FLUOS /PI double-stained cells for apoptosis were quantitatively assayed on a FACSCAN flow cytometer (Becton Dickinson, San Jose, CA, USA) using CellQuest software. The results detected by flow cytometry are shown as annexin V-FLUOS /PI plots. Apoptotic cells are indicated in right upper-lower quadrant in each plot.

### 4.5. Immunoblot Assay

After AF/AD treatment in the absence or presence of SB203580 or 3-MA, cells were lyzed in buffer solution containing 25 mM 4-(2-hydroxyethyl)-1-piperazineethanesulfonic acid (pH 7.5), 0.3 M NaCl, 1.5 mM MgCl_2_, 0.2 mM ethylenediaminetetraacetic acid, 0.1% Triton X-100, 20 mM β-glycerophosphate, 0.5 mM dithiothreitol, 1.0 mM sodium orthovanadate, 0.1 mM okadaic acid, and 1.0 mM phenylmethylsulfonyl fluoride. Equal amounts of lysate were resolved by sodium dodecyl sulfate-polyacrylamide gel electrophoresis (SDS-PAGE) and transferred to polyvinylidene fluoride membranes. After blocking, the blots were incubated with specific primary antibodies (anti-phos-P38MAPK, anti-Atg5, anti-Atg7, anti-Atg10, anti-Atg12, Anti-Beclin-1, and anti-LC3 antibodies) overnight at 4 °C and further incubated for 1 h with horseradish peroxidase-linked secondary antibodies. Proteins were visualized using an enhanced chemiluminescence kit with Lumino Image Analyzer (Founder, Beijing, China). All densitometric quantifications of protein levels were made relative to β-Actin and expressed in arbitrary units.

### 4.6. Immunofluorescence Assay of Beclin-1 and LC3

After AF/AD treatment in the absence or presence of SB203580 or 3-MA, cells were incubated for 1 h with a 1:500 dilution of specific primary antibody (anti-Beclin-1, anti-LC3,) and further incubated for 1 h with a 1:2000 dilution of goat anti-mouse IgG(H+L)-FITC (for Beclin-1), rabbit anti-goat IgG(H+L)-TRITC (for LC3) as a secondary antibody. Fluorescence in cells was observed under a DY5000X fluorescence microscope (Chongqing Photoelectric Instrument Co. Ltd., Chongqing, China).

### 4.7. JC-1 Assay for ΔΨm

The loss of ΔΨm was detected by JC-1 assay using fluorescence microscopy and flow cytometry. For fluorescence microscopy analysis, after treatment with AF/AD in the absence or presence of s SB203580 or 3-MA, cells were incubated in culture medium containing 2.5 μg/mL JC-1 for 20 min, and then analyzed using a DY5000X fluorescence microscope (Chongqing Photoelectric Instrument Co. Ltd., Chongqing, China). For flow cytometry analysis, after treatment with AF/AD in the absence or presence of SB203580 or 3-MA, cells were incubated in PBS containing 2.5 μg/mL JC-1 for 20 min. Approximately 1 × 10^4^ cells were detected on a FACSCAN flow cytometer (Becton Dickinson, Franklin Lakes, NJ, USA) using CellQuest software. Dotplots presents the results detected by flow cytometry. The shift down of fluorescence from red to green indicates the loss of the ΔΨm in each plots.

### 4.8. Statistical Analyses

All data are means ± S.D. from three independent experiments, and analyzed using an analysis of variance (ANOVA). *p* value less than 0.05 was considered significant and *P* value less than 0.01 was considered highly significant in all cases. SPSS 18.0 software (SPSS Inc., Chicago, IL, USA) was use to perform statistical analyses.

## 5. Conclusions

In the present study, we found that AF/AD resulted in apoptosis concomitant with autophagy, upregulation of p-P38MAPK, and autophagy-associated genes proteins (Atg5-Atg12 protein complex, Atg7, Atg10, Beclin-1, LC3 I, and LC3 II) and downregulation of ΔΨm; that SB203580 attenuated the effects of AF/AD; and that 3-MA inhibited the impact of AF/AD on autophagy, Atg5-Atg12 protein complex, Atg7, Atg10, Beclin-1, LC3 I, LC3 II, and ΔΨm, and promoted the influence of AF/AD on apoptosis and p-P38MAPK in Bel-7402 cells. Taken together, we conclude that crosstalk between P38MAPK and autophagy regulates mitochondria-mediated apoptosis induced by AF/AD in Bel-7402 cells.
